# Supplementary material for: KIN10 promotes stomatal development through stabilization of the SPEECHLESS transcription factor
Source: Nat Commun. 2020 Aug 25;11:4214. doi: 10.1038/s41467-020-18048-w (PMC7447634; doi:10.1038/s41467-020-18048-w)
Supplement: Supplementary file 1 — Supplementary Information [file 41467_2020_18048_MOESM1_ESM.pdf]

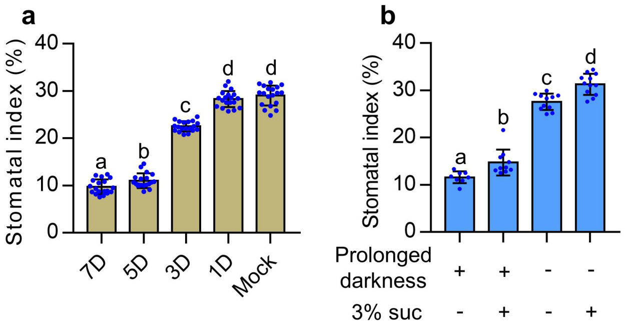

Supplementary Fig. 1. Quantification of the effects of prolonged darkness and exogenous sucrose on the stomatal development.

a, The prolonged darkness inhibited the stomatal development on the abaxial cotyledons of 10-day-old wild type plants. Seedlings of wild type Col-0 were grown on ½ MS solid medium without sucrose under long-day condition for 10 days or transferred to darkness different days (n=20). **b**, Sucrose supply partially suppressed the decreased stomatal index by the prolonged darkness. Seedlings of wild type Col-0 were grown on ½ MS solid medium with or without 3% sucrose under long-day condition for 3 days and then transferred to darkness for 7 days, or continuously under long-day condition for 10 days (n=10). Error bars means the S.D.. Different letters above the bars indicated statistically significant differences between the samples (ANOVA analysis followed by Uncorrected Fisher's LSD multiple comparisons test, $p < 0.05$).

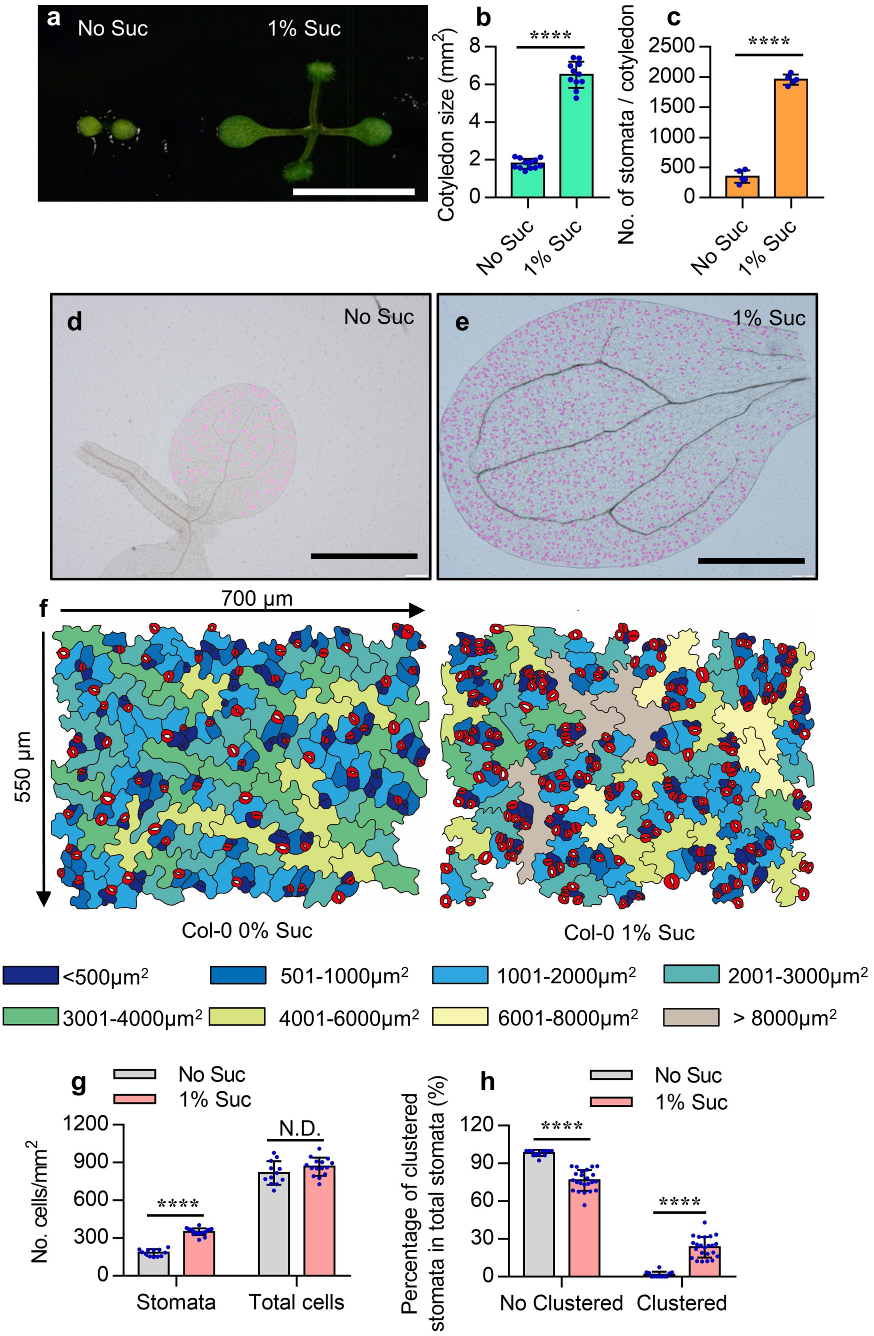

a
No Suc
1% Suc
b
Cotyledon size (mm2)
8
6
4
2
0
****
No Suc
1% Suc
c
No. of stomata / cotyledon
2500
2000
1500
1000
500
0
****
No Suc
1% Suc
d
No Suc
e
1% Suc
f
700 µm
550 µm
Col-0 0% Suc
Col-0 1% Suc
<500µm2
501-1000µm2
1001-2000µm2
2001-3000µm2
3001-4000µm2
4001-6000µm2
6001-8000µm2
> 8000µm2
g
No Suc
1% Suc
No. cells/mm2
1200
900
600
300
0
****
N.D.
Stomata
Total cells
h
No Suc
1% Suc
Percentage of clustered stomata in total stomata (%)
120
90
60
30
0
****
****
No Clustered
Clustered

Supplementary Fig. 2. Sucrose regulates the leaf development in Arabidopsis.

a, Seedling images of Arabidopsis wild type plants (Col-0) grown in ½ MS liquid medium with or without 1% sucrose under long-day condition for 10 days. Scale bars represent 1 cm. **b-c,** Quantification of cotyledon size **(b)** (n=10) and numbers of stomata in a whole cotyledon **(c)** (n=5) on Arabidopsis Col-0 plants grown in ½ MS liquid medium with or without 1% sucrose. **d-e,** DIC images of abaxial cotyledons of Col-0 plants grown in ½ MS liquid medium with or without 1% sucrose for 10 days. Stomata on cotyledons are false colored in pink for easier identification. Scale bars represent 1 mm. **f,** Abaxial epidermis of cotyledons of Col-0 plants grown in ½ MS liquid medium with or without 1% sucrose for 10 days. Cell size distribution is presented as a colour scale. **g-h**, Quantification of the effects of sucronse on the stomata density and the ratio of clustered stomata. Seedlings of wild type were grown in ½ MS liquid medium with or without 1% sucrose for 10 dyas under long-day condition. Error bars indicate standard deviation (s.d.) (n=12). (ANOVA analysis followed by Uncorrected Fisher's LSD multiple comparisons test, *$p < 0.05$, **$p < 0.01$,****$p<0.0001$).

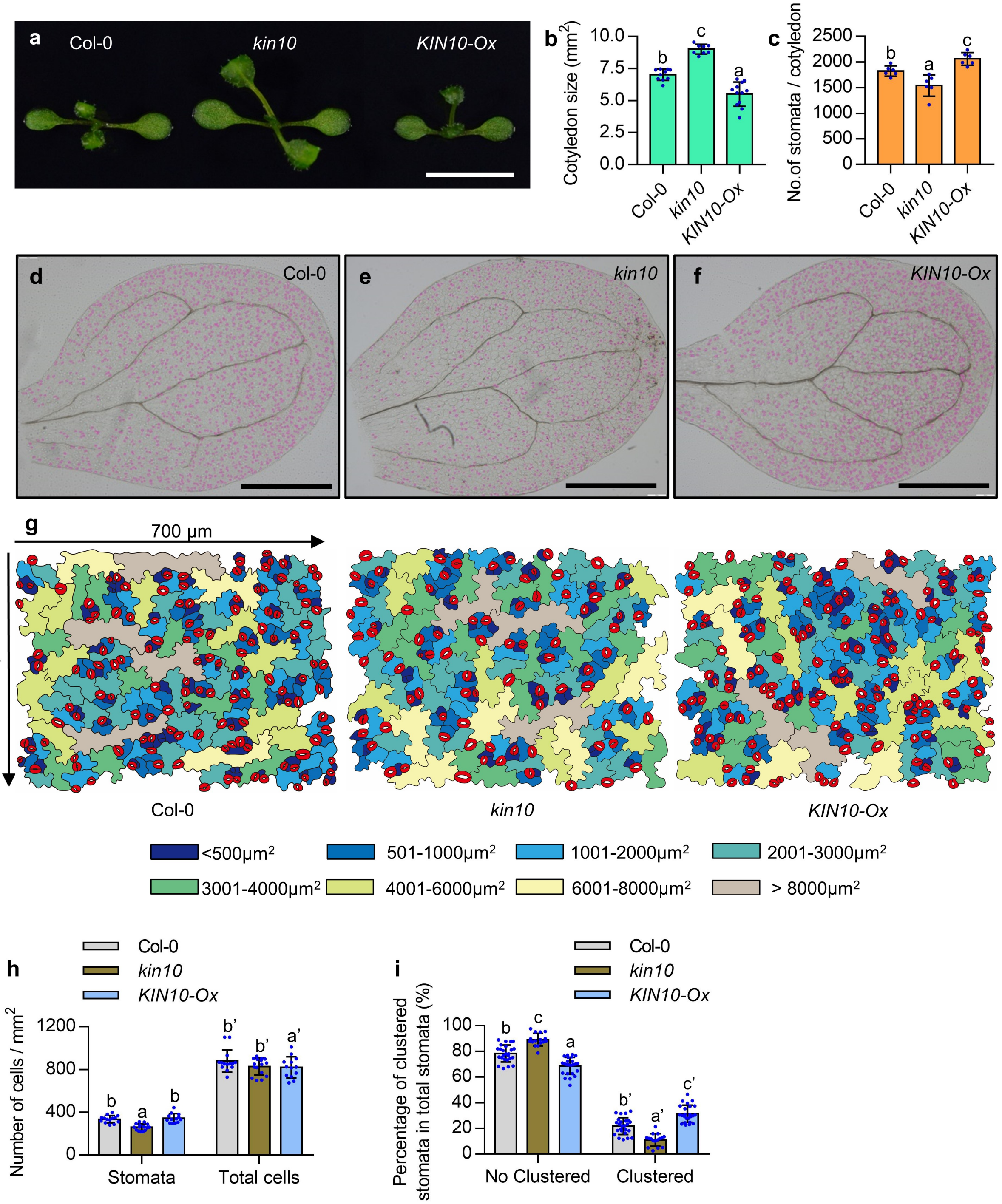

a
Col-0
kin10
KIN10-Ox
b
Cotyledon size (mm2)
10.0
7.5
5.0
2.5
0.0
b
c
a
Col-0
kin10
KIN10-Ox
c
No.of stomata / cotyledon
2500
2000
1500
1000
500
0
b
a
c
Col-0
kin10
KIN10-Ox
d
Col-0
e
kin10
f
KIN10-Ox
g
700 µm
550 µm
Col-0
kin10
KIN10-Ox
<500µm2
501-1000µm2
1001-2000µm2
2001-3000µm2
3001-4000µm2
4001-6000µm2
6001-8000µm2
> 8000µm2
h
Col-0
kin10
KIN10-Ox
Number of cells / mm2
1200
800
400
0
b
a
b
b'
b'
a'
Stomata
Total cells
i
Col-0
kin10
KIN10-Ox
Percentage of clustered stomata in total stomata (%)
100
80
60
40
20
0
b
c
a
b'
a'
c'
No Clustered
Clustered

Supplementary Fig. 3. KIN10 promotes stomatal development.

a, Seedling images of wild type plants (Col-0), *kin10* and *KIN10-Ox (p35S::KIN10-myc-2#)* that were grown in ½ MS liquid medium containing 1% sucrose under long-day condition for 10 days. Scale bar represent 1 cm. **b-c,** Quantification of cotyledon size **(b)** (n=8) and amount of stomata in the whole cotyledon **(c)** (n=6) of Col-0, *kin10* and *KIN10-Ox* plants. Different letters above the bars indicated statistically significant differences between the samples (ANOVA analysis followed by Uncorrected Fisher's LSD multiple comparisons test, $p < 0.05$). **d-f,** DIC images of entire 10-d-old abaxial cotyledons of Col-0 **(d)**, *kin10* **(e)** and *KIN10-Ox* **(f)** plants grown in ½ MS liquid medium containing 1% sucrose under long-day for 10 days. Stomata on cotyledons are false colored in pink for easier identification. Scale bar represent 1 mm. **g,** Abaxial epidermis of cotyledons of wild-type Col-0, *kin10*, *KIN10-Ox* that were grown in ½ MS liquid medium containing 1% sucrose for 10 days. Cell size distribution is presented as a color scale. **h,** Quantification of stomata density of 10-day-old Col-0, *kin10*, *KIN10-Ox* plants (n=14). **i,** Ratio of clustered stomata on Col-0, *kin10*, *KIN10-Ox* plants (n=16). Error bars mean S.D. Different letters above bars indicates statistically significant differences between the samples (ANOVA analysis followed by Uncorrected Fisher's LSD multiple comparisons test, $p < 0.05$).

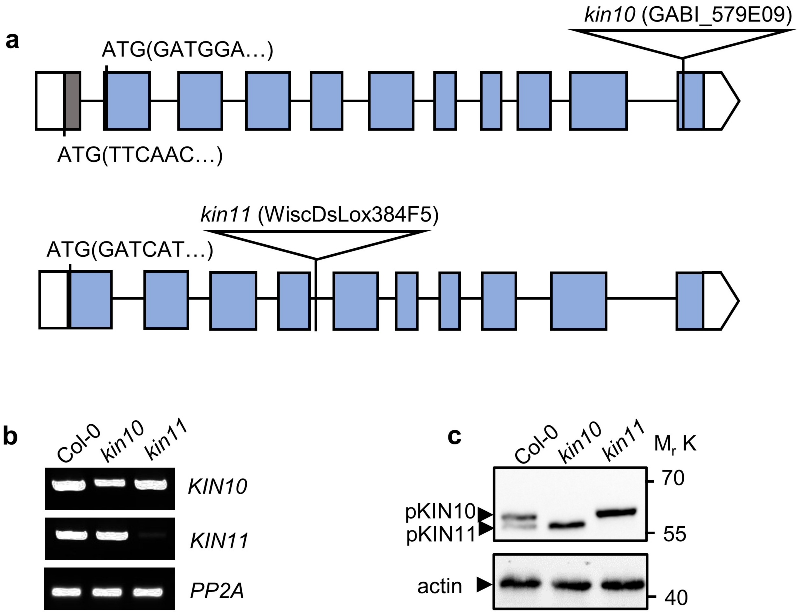

Supplementary Fig. 4. T-DNA insertion sites and expression levels of KIN10 and KIN11 in their corresponding signal mutants.

a, Schematic diagram showing the mutagenesis or T-DNA insertion site in the *KIN10* and *KIN11* genomic region. White boxes: 5' UTR, grey boxes: Splicing exon, blue boxes: exon, arrows: 3' UTR, lines: intron. **b**, RT-PCR analysis the expression of *KIN10* and *KIN11* in wild type and mutants. *PP2A* was used as the internal control. **c**, Immunoblot analysis the phosphorylated KIN10 or KIN11 in the wild type and mutants. The phosphorylated KIN10 or KIN11 proteins were analyzed by anti-AMPKT172 antibody. Actin bands were used as loading control.

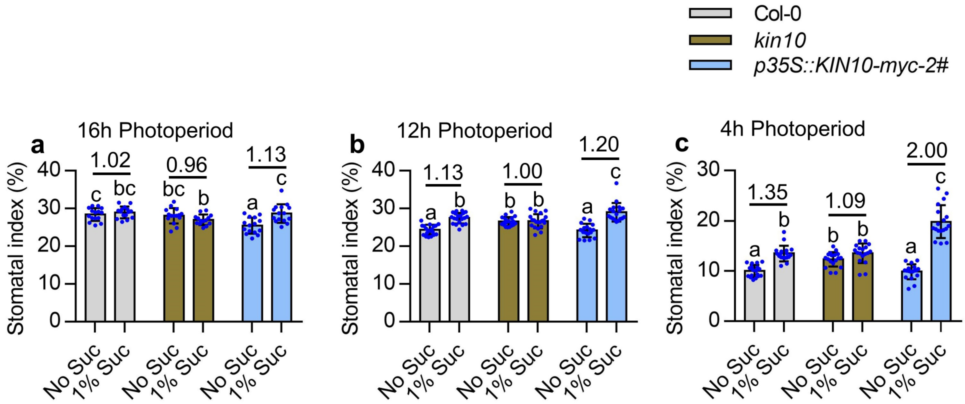

Supplementary Fig. 5. KIN10 is a positive regulator for stomatal development under certain conditions.

a-c, Quantification of the effects of KIN10 on the stomatal development under different photoperiod conditions. Seedlings of Col-0, *p35S:KIN10-myc-2#* and *kin10* were grown on ½ MS solid medium with or without 1% sucrose for 10 days under 16h light/8h dark photoperiod (**a**), 12h light/12h dark photoperiod (**b**), and 4h light/20h dark photoperiod conditions (**c**). Error bars indicate standard deviation (S.D.) (n=20). Numbers between bars indicated the relative fold changes of average means in the indicated materials. Different letters above the bars indicated statistically significant differences between the samples (ANOVA analysis followed by Uncorrected Fisher's LSD multiple comparisons test, $p < 0.05$).

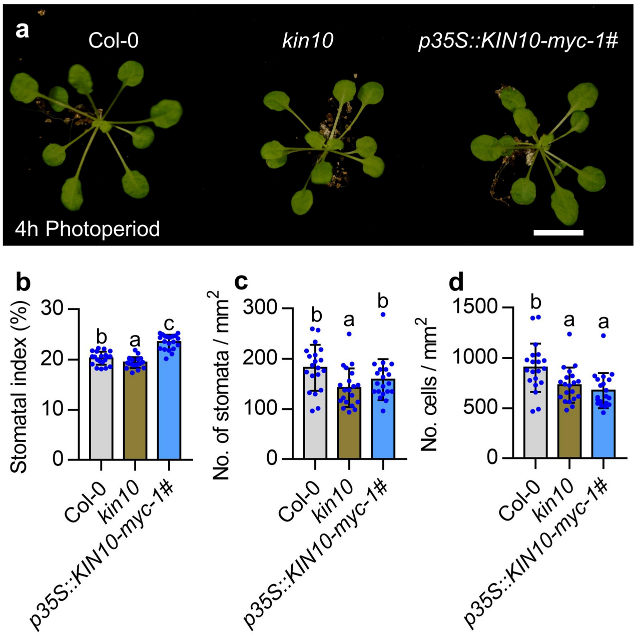

Supplementary Fig. 6. KIN10 promotes the stomatal development in rosette leaves.

a, Representative plants of wild type Col-0, *p35S:KIN10-myc-1#* and *kin10* grown in soil under 4h light/20h dark photoperiod for 5 weeks. Scale bar represent 1 cm. **b-d**, Quantification of stomatal index (**b**), stomatal density (**c**) and cell density (**d**) in the 5th rosette leaves of wild type Col-0, *p35S:KIN10-myc-1#* and *kin10* that were grown in soil under 4h light/20h dark photoperiod for 5 weeks. Error bars indicate standard deviation (S.D.) (n=20). Different letters above the bars indicated statistically significant differences between the samples (ANOVA analysis followed by Uncorrected Fisher's LSD multiple comparisons test, $p < 0.05$).

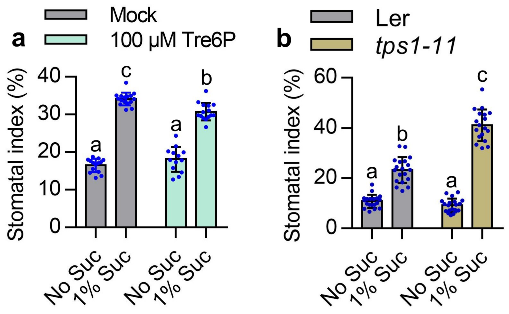

Supplementary Fig. 7. Tre6P negatively regulates stomatal development.

a, Quantification of the effects of Tre6P on stomatal index in wild type abaxial cotyledons. Seedlings of wild type Col-0 were grown in the liquid ½ MS medium containing Tre6P and 1% sucrose or 1% sucrose only for 10 days under 16h light/8h dark photoperiod (n=14) 100μM. **b**, Mutation TPS1 resulted in the increased stomatal index. Seedlings of wild type Ler and *tps1-11* were grown in the liquid ½ MS medium containing 1% sucrose for 10 days under 16h light/8h dark photoperiod (n=20). Error bars indicate standard deviation (S.D.). Different letters above the bars indicated statistically significant differences between the samples (ANOVA analysis followed by Uncorrected Fisher's LSD multiple comparisons test, $p < 0.05$).

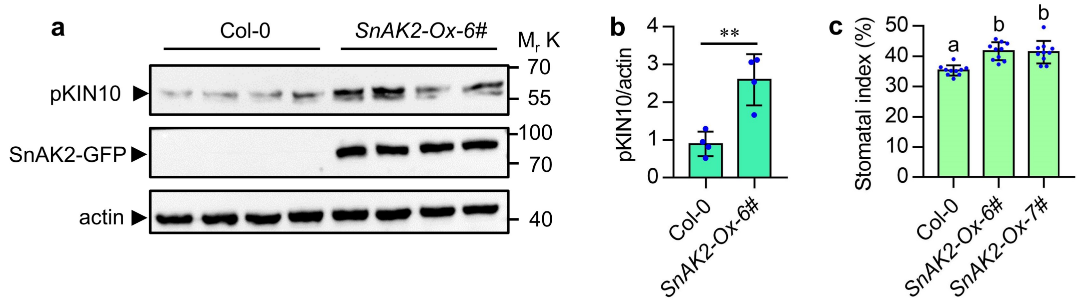

Supplementary Fig. 8. Overexpression of *SnAK2* led to the increased stomatal index.

a, Immunoblot analysis of phosphorylated KIN10 proteins in wild type Col-0 and SnAK2-Ox plants. Seedlings of Col-0 *and p35S:SnAK2-GFP* (*SnAK2-Ox*) were grown in ½ MS liquid medium under long-day condition for 3 days. The phosphorylated KIN10 proteins were analyzed by anti-AMPKT172 antibody, SnAK2-GFP were probed with anti-GFP antibody. Actin bands were used as loading control. **b,** Quantification analysis of the levels of phosphorylated KIN10 protein in wild type and SnAK2-Ox plants. The ratio of phosphorylated KIN10 to actin was quantified by ImageJ software. Error bars mean the S.D. (n = 4). Asterisk above dots indicates significant accumulation of phosphorylated KIN10 protein by SnAK2 overexpression (Student t-test, ***p < 0.01*). **c,** *SnAK2-Ox* lines performed higher stomatal index in cotyledon than wild type Col-0 seedlings grown on ½ MS liquid culture containing 1% sucrose for 10 days.

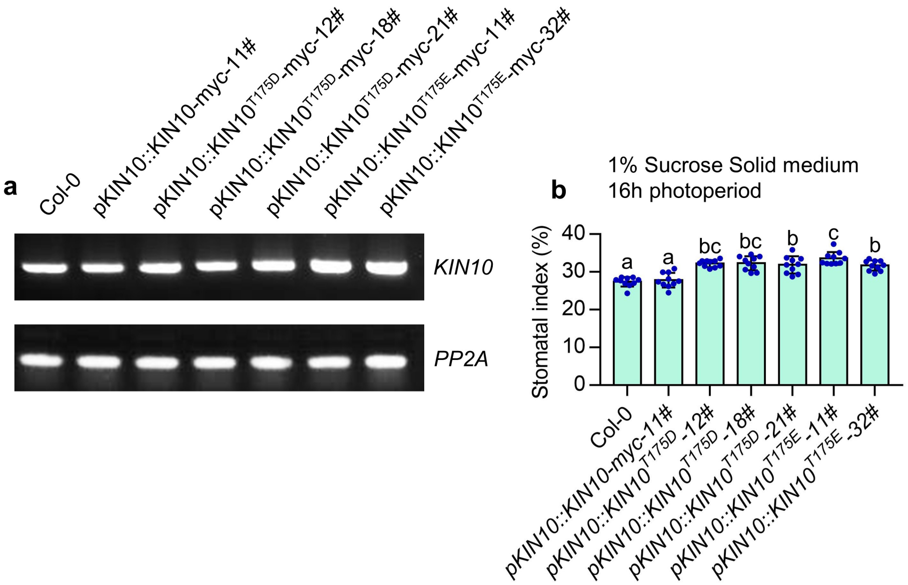

Supplementary Fig. 9. Mutation of Thr175 to Asp or Glu enhances the promoting effects of KIN10 on stomatal development

a, Semi-quantitative RT-PCR analysis the expression levels of *KIN10-myc* and *KIN10*^{T175D}*–myc* in wild type and different transgenic plants. Seedlings of Col-0, *pKIN10:KIN10-myc* and *pKIN10:KIN10*^{T175D}*-myc* transgenic plants were grown on solid medium with 1% sucrose under 16h light/8h dark for 5 days. *PP2A* was used to as the internal control. **b**, Site directed mutation of Thr175 to aspartic acid enhances the promoting effects of KIN10 on stomatal development. Seedlings of Col-0*, pKIN10:KIN10-myc* and *pKIN10:KIN10*^{T175D}*-myc* transgenic plants were on solid medium with 1% sucrose under 16h light/8h dark for 10 days. Error bars indicate standard deviation (S.D.) (n=10). Different letters above the bars indicated statistically significant differences between the samples (ANOVA analysis followed by Uncorrected Fisher's LSD multiple comparisons test, $p < 0.05$).

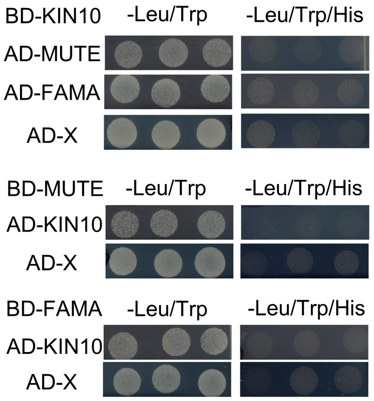

Supplementary Fig. 10. KIN10 did not interact with MUTE and FAMA in yeast.

Yeast-two hybrid (Y2H) assay showed that KIN10 had no interaction with MUTE and FAMA.

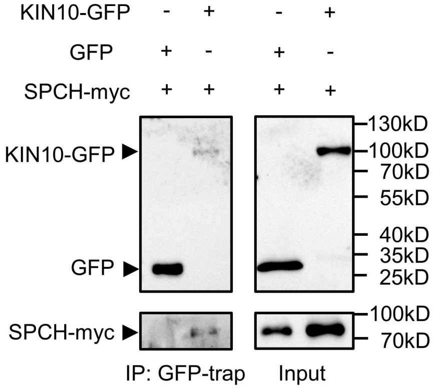

Supplementary Fig. 11. KIN10 interacted with SPCH in plants.

Coimmunoprecipitation assays showed KIN10 interacted with SPCH in plant cells. The protoplasts prepared from Arabidopsis mesophyll cells were used to cotransform *p35S:KIN10-GFP* and *p35S:SPCH-myc* constructs or *p35S:GFP* and *p35S:SPCH-myc* constructs. Immunoprecipitation was performed using GFP trap and immunoblots were probed with anti-myc or anti-GFP antibodies.

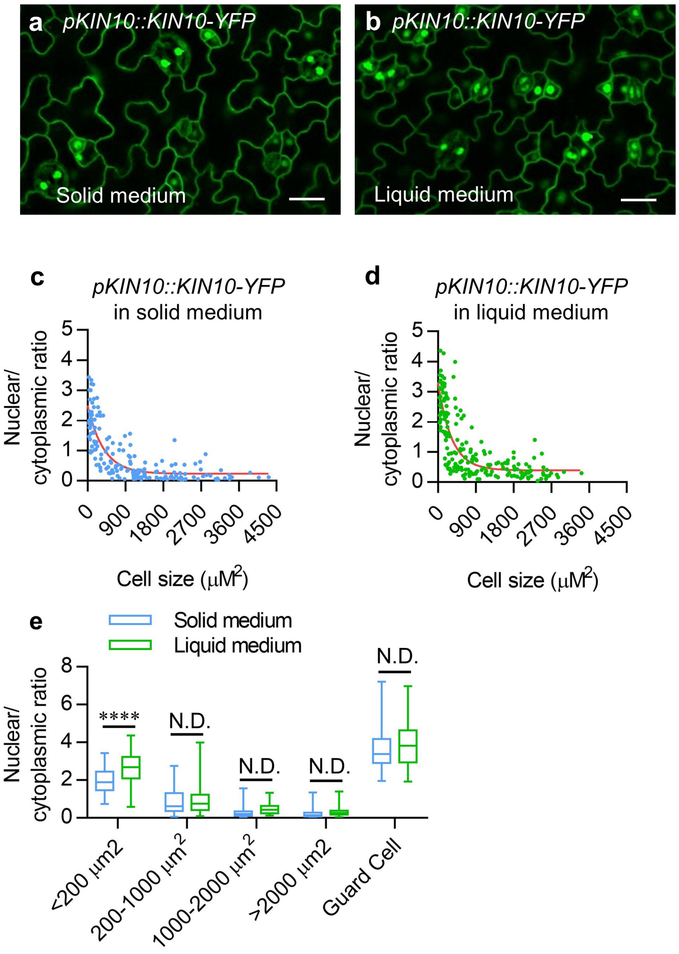

Supplementary Fig 12. Nuclear-localized KIN10 performed the same pattern in both 1/2 MS liquid medium or solid medium containing 1% sucrose. a-b, The subcellular location of pKIN10:KIN10-YFP in Arabidopsis epidermal leaves. Seedlings of pKIN10:KIN10-YFP were grown in ½ MS liquid medium (**a**) or in ½ MS solid medium (**b**) containing 1% Sucrose for 5 days under long-day condition Scale bars in confocal images represent 20 μm. **c-e**, Quantification of nuclear localization of KIN10 in different scale epidermal cells of pannel (**a**) and pannel (**b**), respectively. Nuclear and cytoplasmic KIN10-YFP signal from more than 200 epidermal cells in 10 cotyledons were analyzed by ImageJ software. Scatter plot **(c** and **d)** and box plot **(e)** showed negative relationship of KIN10-YFP nuclear/cytoplasmic ratio and the size of epidermal cells. Error bars indicate standard deviation (S.D.). Different letters above the bars indicated statistically significant differences between the samples (ANOVA analysis followed by Uncorrected Fisher's LSD multiple comparisons test, $p < 0.05$).

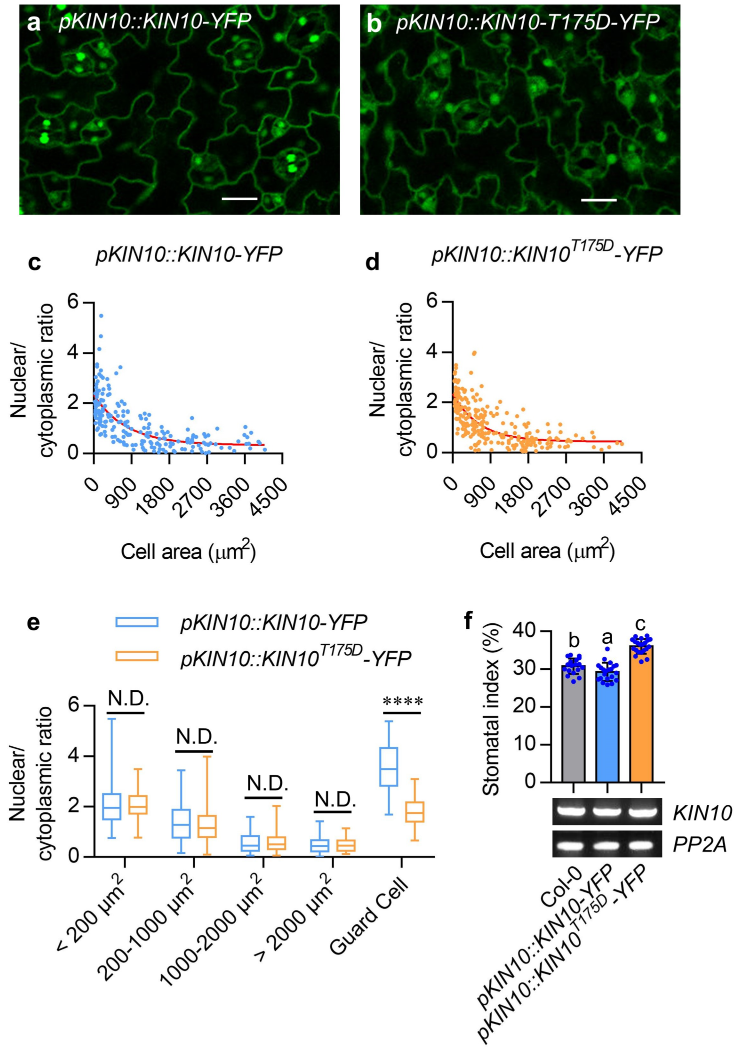

Supplementary Fig 13. The T-loop phosphorylation had no significant effects on the subcellular location of KIN10-YFP in stomatal cells.

a-b, The subcellular location of *pKIN10:KIN10-YFP* and *pKIN10:KIN10^{T175D}-YFP* in Arabidopsis epidermal leaves. Seedlings of *pKIN10:KIN10-YFP* (**a**) and *pKIN10:KIN10^{T175D}-YFP* (**b**) were grown in ½ MS Solid medium containg 1% Sucrose for 5 days under long-day condition. Scale bars in confocal images represent 20 μm. **c-e**, Quantification of nuclear localization of KIN10-YFP and KIN10^{T175D} in different scale epidermal cells of pannel (**a**) and pannel (**b**), respectively. Nuclear and cytoplasmic KIN10-YFP or KIN10^{T175D}-YFP signals from more than 200 epidermal cells in 10 cotyledons were analyzed by ImageJ software. Scatter plot **(c** and **d)** and box plot **(e)** showed negative relationship of KIN10-YFP nuclear/cytoplasmic ratio and the size of epidermal cells. Asterisk between bars indicated statistically significant differences between the samples (Student's t-test, ****$p < 0.0001$). Error bar indicates S.D. **f**, Quantification of the stomatal index of wild type, *pKIN10:KIN10-YFP* and *pKIN10:KIN10^{T175D}-YFP*. Seedlings were grown in ½ MS solid medium containing 1% Sucrose for 10 days under long-day condition. RT-PCR showed KIN10 expression are equal among different transgenic lines. Error bars indicate standard deviation (S.D.). Different letters above the bars indicated statistically significant differences between the samples (ANOVA analysis followed by Uncorrected Fisher's LSD multiple comparisons test, $p < 0.05$).

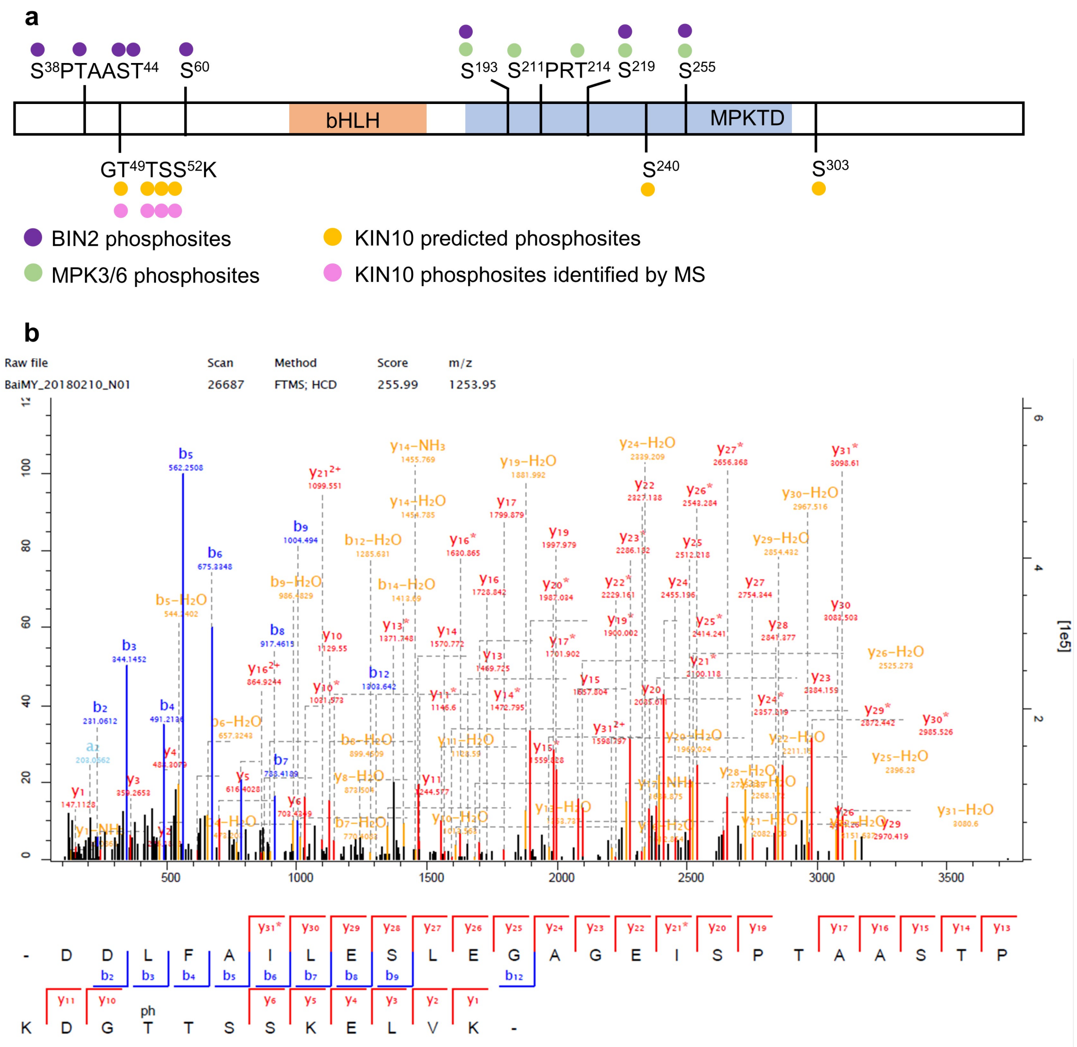

Supplementary Fig. 14. KIN10 phosphorylation sites on SPCH protein.

a, Scheme of the SPCH protein indicating KIN10 phosphorylation sites. The red dots mark *in vitro* KIN10 phosphorylation targets identified by mass spectrometer (this study), yellow dots designate the predicated KIN10 phosphorylation residues based on KIN10 conserved motif, the purple dots refer to the previously identified BIN2 phosphorylated residues, the green dots mean the previously identified MPK3 and MPK6 phosphorylated residues. **b,** Mass spectrometry analysis of KIN10 phosphorylation sites on SPCH.

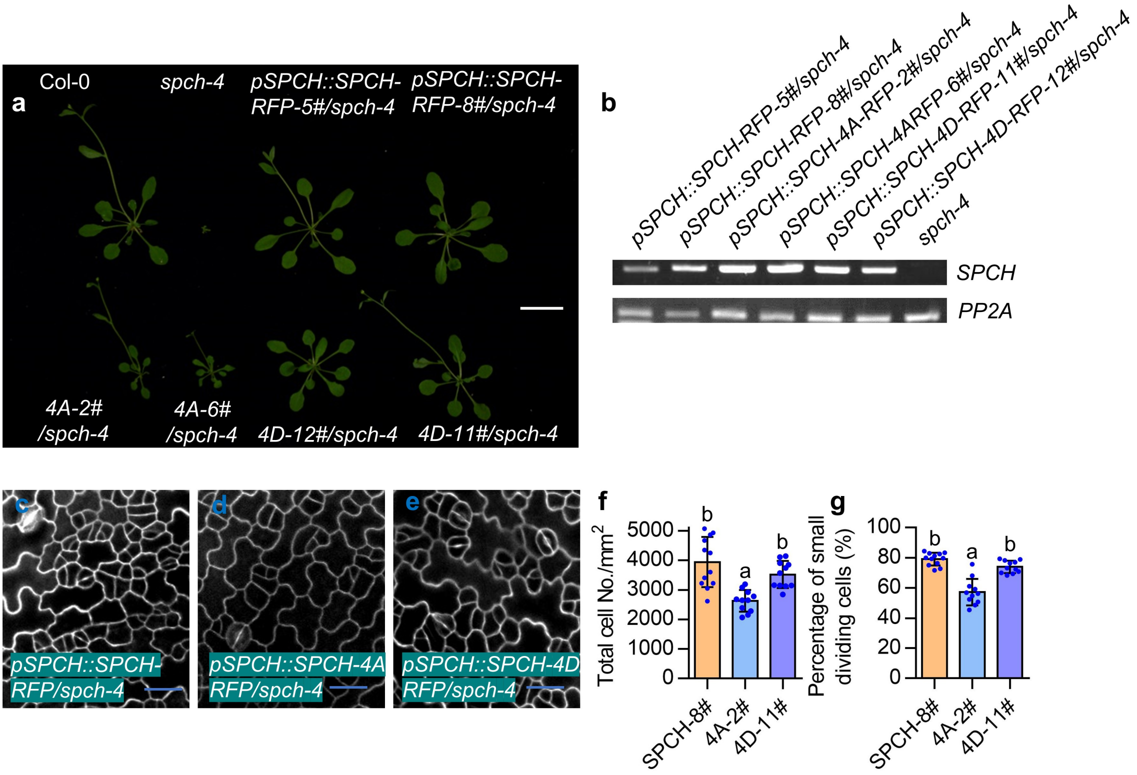

Supplementary Fig. 15. Functional analysis of KIN10 driving phosphosites on SPCH.

a, Phenotype of mutated SPCH transgenic plants grown in soil for 4 weeks under long-day condition. Scale bar, 2 cm. **b**, RT-PCR analysis the expression levels of *SPCH* and mutation form of *SPCH* in *spch-4* and different transgenic plants. *PP2A* was used to verify equal cDNA loading. **c-g**, Quantification of total cells density and dividing small cells ratio in abaxial cotyledons of 3 days old different SPCH-related transgenic plants. Seedlings were grown on ½ MS medium containing 1% sucrose under long-day condition for 3 days. Different letters above the bars indicated statistically significant differences between the samples (ANOVA analysis followed by Uncorrected Fisher’s LSD multiple comparisons test, $p < 0.05$). Error bar indicates S.D. (n=10). PI-marked cell outlines are in white. Scale bars in confocal images represent 20 μm.

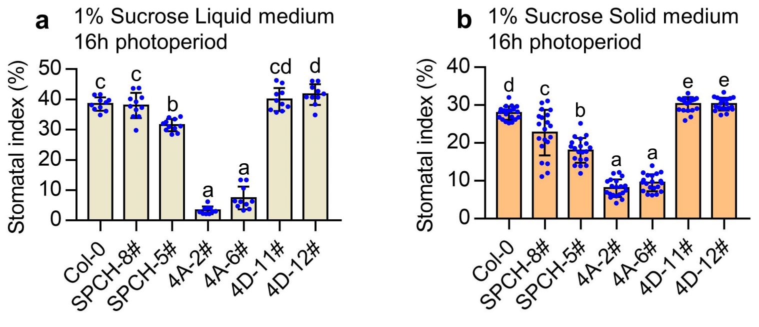

Supplementary Fig. 16. The KIN10-phosphorylation sites are critical for SPCH to promote stomatal development.

a-b, Quantification of the effects of different mutant versions of SPCH on stomatal development. Seedlings of wild type, *pSPCH::SPCH-RFP/spch-4, pSPCH:SPCH4A-RFP/spch-4* and *pSPCH:SPCH4D-RFP/spch-4* were grown in ½ MS liquid medium (**a**) or solid medium (**b**) containing 1% Sucrose for 10 days under 16h photoperiod condition. Error bars means the S.D. (n=10-20). Different letters above the bars indicated statistically significant differences between the samples (ANOVA analysis followed by Uncorrected Fisher's LSD multiple comparisons test, $p < 0.05$).

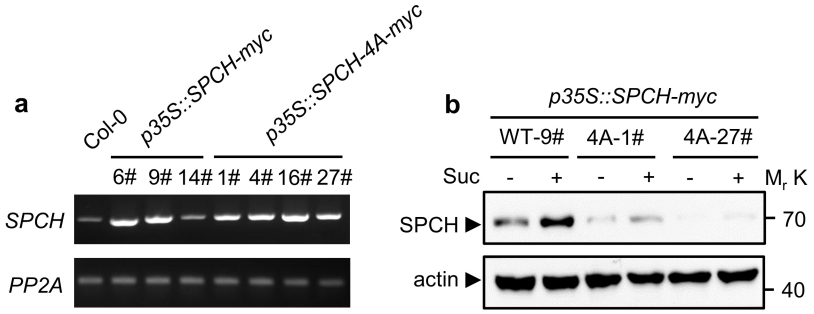

Supplementary Fig. 17. Sucrose increases the SPCH protein stability.

a, RT-PCR analysis the expression levels of *SPCH* and mutation form of *SPCH* in wild type and different transgenic plants. *PP2A* was used to verify equal cDNA loading. **b,** Immunoblot analysis of the effects of sucrose on the protein levels of SPCH-myc and SPCH4A-myc. Seedlings of *p35S::SPCH-myc* and *p35S:SPCH4A-myc* were grown in sugar free medium for 3 days, and then treated with 1% sucrose for 24 hours. Total SPCH proteins were probed with anti-myc antibody, actin bands were used as loading control. An antibody against actin was used to verify equal protein loadings.

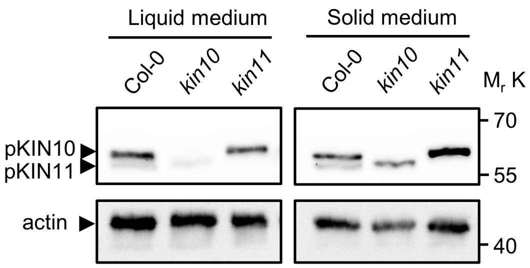

Supplementary Fig.18. Immunoblot analysis the phosphorylated KIN10 and KIN11 in wild type, *kin10* and *kin11* mutants.

Seedlings of wild type, *kin10* and *kin11* mutant were grown under 12h light/12 dark for 10 days in liquid sugar free medium or on solid sugar free medium. The phosphorylated KIN10 proteins or KIN11 proteins were analyzed by anti-AMPKT172 antibody, actin bands were used as loading control.

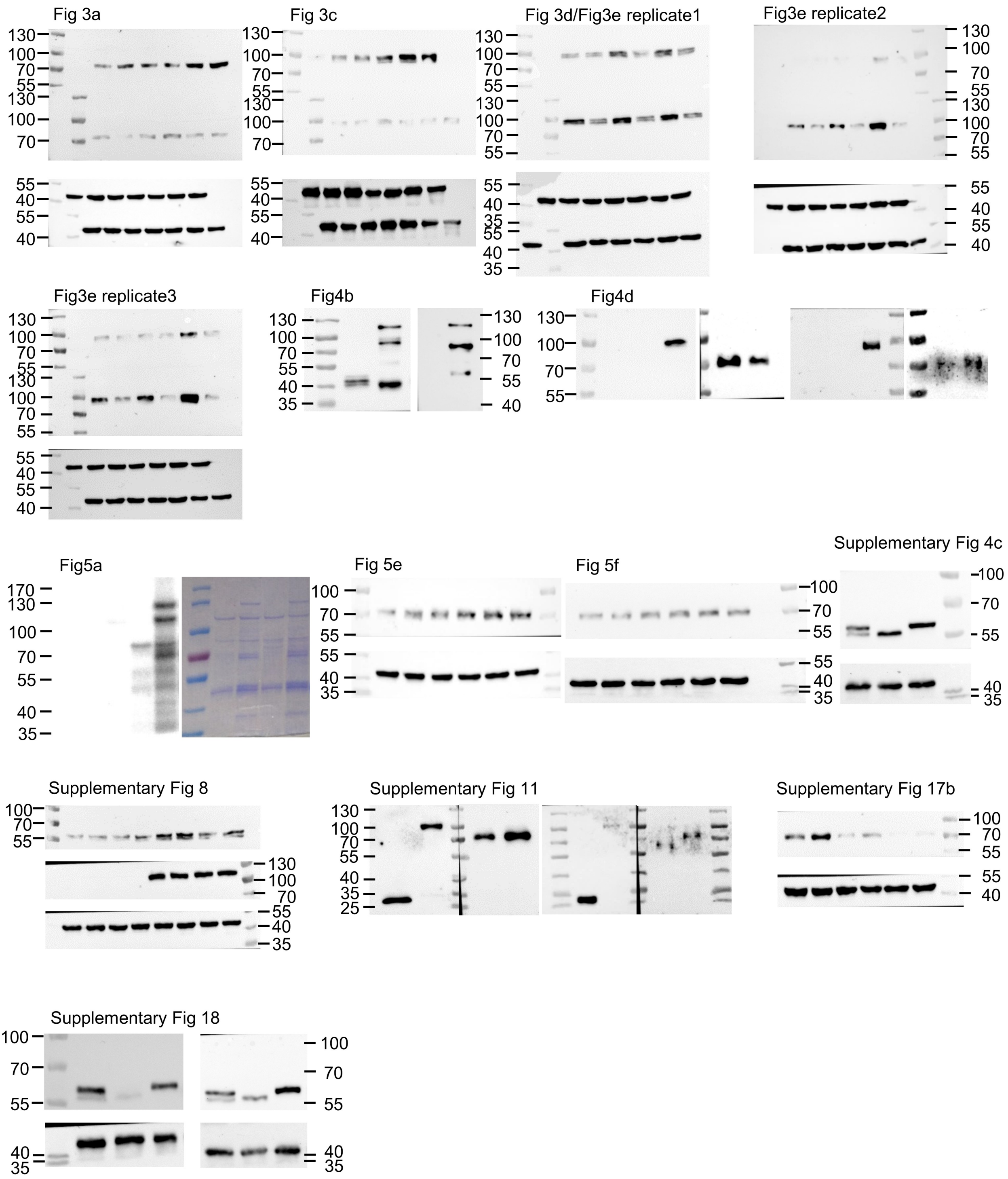

Supplementary Fig. 19. Full scan data of original immunoblots.

Supplementary Table 1. Primers used in this study

Function	name	Sequence
KIN10 CDS clone	KIN10 Fwd	ATGTTCAAACGAGTAGATGAGTTTAATTTAGTT
	KIN10 Rev	GAGGACTCGGAGCTGAGCA
KIN10 promoter and genomic	KIN10 gFwd	TATTGAGGTTTCCGGAATTCCCTT
	KIN10 Rev	GAGGACTCGGAGCTGAGCAAGA
SnAK1 CDS clone	SnAK1 Fwd	ATGTTTCGTGATAGTTTTTGTTTGCCCG
	SnAK1 Rev	TTAGTTAGGATCTGAGGTTTCAGAGATCC
SnAK2 CDS clone	SnAK2 Fwd	ATGTTTTGTGATAGTTTTGCATTTGC
	SnAK2 Rev	GCTATGGTTTTGATCTTCTTCTTCTTC
SPCH CDS	SPCH Fwd	ATGCAGGAGATAATACCGGATTTT
	SPCH Rev	CAAGCAGAATGTTTGCTGAATTTGTT
SPCH promoter	pSPCH Fwd	GAGCTCATCATCACTGCGATAAGGAG
	pSPCH Rev	ACTAGTCGTGATTAGAGATATATCCTT CTCTCTCTCTC
KIN10 qRT-PCR	qKIN10 Fwd	CAACCGAACCCAGAATGATGGC
	qKIN10 Rev	AACCACTAGAGGCACGGAAACG
TPS5 qRT-PCR	qTPS5 Fwd	AGCTTATGGAACACCTCGAAAGCG
	qTPS5 Rev	GACCTTTGTTCACACCCTGTGG
PP2A qRT-PCR	qPP2A Fwd	TTCTCGCTCCAGTAATGGGATCCGA
	qPP2A Rev	GTTCTCCACAACCGCTTGGTCGACT
SPCH RT-PCR	rtSPCH Fwd	ATGCAGGAGATAATACCGGATTTT
	rtSPCH Rev	CCTTCACCGCCTGTTCTAAG
KIN10 RT-PCR	rtKIN10 Fwd	CAACCGAACCCAGAATGATGGC
	rtKIN10 Rev	GAGGACTCGGAGCTGAGCA
KIN11 RT-PCR	rtKIN11 Fwd	ATGGATCATTCATCAAATAGATTTGGC
	rtKIN11 Rev	CGTTTCACCGGGTCAACTATAAGCATC
